# Monthly Summary

**Published:** 1879-07

**Authors:** 


### MONTHLY SUMMARY.

---

*The Woman-Doctor Question.*—It is sometimes stated that the woman doctor is a recognized institution in the empire of the Czar, a belief in some sort warranted by the prominence which has of late years been given in the medical schools of St. Petersburg to the training of women students in physic. According to the Russian *Medical Gazette*, it would appear that the question of women medical practitioners is not yet settled in Russia. On the 29th of October last the right of women who had completed a course of medical training to practice medicine in the empire was brought formally under the consideration of the Sanitary Council attached to the Ministry of the Interior. After prolonged discussion, the Council unanimously resolved, as follows:—" Although the right to practice medicine by the female students of the medical faculty has not to the present been recognized by the legislative authority, having regard to the evidence now submitted by the professors, that these students are fully competent to exercise the medical profession, the Sanitary Council will itself endeavor to obtain from the Government the authorization necessary for them to enter upon practice." Forty-eight American ladies, students of medicine, under the guidance of a professor, have recently visited the hospitals and scientific establishments of Venice, Milan, Florence and Parma. —*Med. and Surg. Reporter.*

---

*How to Avoid Leaving Scars.*—At a conversational meeting of the Philadelphia Medical Society, November 13, 1878, it was addressed by Dr. John H. Packard, on "Some Surgical Wrinkles." The first point he discussed was a method of making superficial incisions by which scarring can be avoided. In operations upon exposed parts, such as the face and hand, it is very desirable that they shall be so done as to leave as little scar as possible. The procedure recommended was first suggested by witnessing the effects of an accident, a lady having fallen while carrying a china dish, a piece of which made a long gaping incised wound in her hand, the sharp, knife-like edge having cut through the skin very obliquely. The wound healed readily, almost without a scar. A few weeks afterwards the traces of the injury could scarcely be discovered.

Thinking that this effect was in great measure due to the direction of the incision through the skin, the speaker tried the experiment in cutting down upon a tumor of the thigh, holding the knife so as to divide the skin obliquely. The wound united perfectly, and after it had healed he actually could not find the line of incision. Since that time he had tested the idea in numerous other cases with highly satisfactory results. In small superficial operations, such as the removal of small tumors from the face, it has a cosmetic advantage that at once recommends it.—*Medical Brief.*

---

*Personal Similarities and Dissimilarities.*—A French writer, M. Delaunay, makes the curious observations that all savages belonging to the same tribe closely resemble each other in form, strength and in intelligence. But in the higher classes of mankind we have a primitive type replaced by multiple and various types of men—individuals differing so much from each other that it is impossible to find two persons absolutely alike. From a sexual point of view, we find more variation among the males than among the females of the same race. This is especially the case in the human species, among the male portion of which there is a much greater diversity of constitution than there is among the female portion. With regard to age, nearly all infants resemble each other, both physically and morally. Moreover, all old men have the same weakness of constitution, the same feelings, the same tastes and the same childish notions. On the other hand, adults present among themselves the greater variations. With regard to constitution, the strong and the intelligent differ from each other much more than the weak and unintelligent.—*Med. and Surg. Reporter.*

---

*Poisoning by Chlorate of Potash.*—This drug is so freely used as a domestic remedy, that the following case, which occurred in the family of Dr. Kauffman, of Berlin, will be read with interest. It is given in the *Med. Central Zeitung:* He used to keep a certain quantity of this salt in a tin box, and give some of it daily to his children, as prophylactic treatment against diptheria, which happened to be epidemic at that time in the neighborhood. One day, the children, while playing, possessed themselves of the box, and took each about half an ounce of the chlorate of potass. The youngest child, a girl two years and a half old, had severe vomiting, which lasted for seven hours, when she died of gastritis, in spite of all help. Another remarkable symptom of the poisoning was the profound lethargy of the child, which probably prevented its showing symptoms of pain. Another similar case is mentioned, of a young man who had taken small doses of chlorate of potass. to cure himself of hoarseness. From the time of taking the first dose to the moment when he left off, the patient suffered from gastritis, and vomited everytime he took the drug. These symptoms ceased as soon as the medicine was discontinued, which clearly shows it to have been the primary cause of the inflammation.—*Med. and Surg. Reporter.*

---

*On the Prevention of Fatal Accidents from Using Anœsthetics.*—Dr. Simonin gives, in the *Revue Med. de l' Est,* 5 année t. x, No. 9, p. 261, the following three observations, which may be considered as very important if attended to. 1. Progressive peripheric insensibility, especially in the temporal region and the cornea. 2. The condition of the muscles and the jaws; the former must be in a complete state of relaxation, and the jaws closed. The adductor muscles of the lower jaw, therefore, form an exception to the rule, by being in a state of trismus. 3. The state of the pupil, which must be contracted, while the respiration becomes more normal, having been much quickened during the stage of excitement. All these phenomena are very important; they are synchronous, and must be carefully observed, as well as respiration and circulation. If the three symptoms cited should not appear coincidently, they must be carefully watched for in various stages of the anæsthesia, because they are sure to oppear at a given moment.—*Med. & Sur. Reporter.*

---

*A New Insect Powder.*—The wild rosemary (*Ledum palustre*) is said to be a first-rate plant for the destruction of all kinds of annoying insects, and may be usefully employed as a substitute for pyrethrum or "Persian insect powder." It can be used dried and pulverised or used fresh. The tincture readily relieves the itching from bites of gnats and mosquitoes. Glycerine added to the tincture and rubbed on the hands and skin is a protection. The plant grows wild in Europe and the northern parts of America, and may be obtained at less cost than the pyrethrum. —*Drug. Circular.*

*Very Large Salivary Calculus.*—Dr. Freudenberg relates the case of a gentleman who consulted him on account of a tumor under the tongue, which gave him only moderate uneasiness, and did not cause any increase of the normal saliva. It dated back six months, and had been since then continually on the increase. On inspection a salivary concretion was found to be projecting to the extent of a centimetre, and by a little manipulation the calculus was dislodged by mere pressure, springing out at last as if from an elastic ring. In length the concretion measured 3.5 centimetres, the diameter of its base measured 1.4 centimetre. It consisted of phoshate and carbonate of lime, and an albuminous substance, the phosphate constituting an unusually large proportion, The calculus occupied the right sublingual gland.—*Berlin Klin. Woch.*

---

*Replanting Teeth.*—A correspondent states that in the preparation of the tooth for replanting or transplanting, he fills the foramen at the end of the root, by inserting in it a gold screw that will completely fill it, then fills pulp chamber and cavity of decay, then upon the surface of the root cuts a groove from apex to neck; this facilitates the escape of the blood as the tooth is placed in the socket.

This, it is alleged, will obviate the pain that otherwise sometimes accompanies the replacement of a tooth.

This groove would rarely, if ever, be required, in transplanting, from the fact that an absolute adaptation of a root to another socket is never found.

Replanting is becoming more and more a favorite method of treating alveolar abscess.—*Dental Register.*

---

*Registration of Dentists in England.*—The law requiring the registration of dentists in the United Kingdom is being enforced, and the officials are overwhelmed with work in that connection. The law applies not only to those exclusively engaged in dental practice, but to those who, engaged in pharmacy, etc., occasionally practice tooth extractions and perhaps perform other operations. The fee is from 2 to 5 pounds sterling, and there are from 1500 to 2000 dentists in practice.

One of the tasks of the Council is the recognition of foreign and colonial dental diplomas, and weighing the evidence entitling applicants to registration. The *Lancet* thinks the task no easy one.

---

*Chronic Pharyngitis,* may be cured, according to a writer in the New York *Medical Journal,* by gargling the throat with water at the temperature of 15° to 20° C. Persistence in the treatment is necessary.

*Ergotine for Neuralgia.*—Marino recommends the following solution as a hypodermic injection in neuralgia: R. Ergotine gr. ijss. to gr iv., distilled water or glycerine q. s., made into a solution for one injection. It causes a more or less intense burning sensation, which disappears in about half an hour, if the part is covered with cold, wet compresses. It does not usually give rise to abscesses or erysipelas, etc. According to the author, a single or at most two injections suffice for a cure, although it is better, in order to prevent a relapse, to give from four to six injections more, according to the intensity of the pain or its long continuance.—*Druggists Circular.*

---

*Treatment of Ulcers.*—Dr. Mandelbaum asserts that ulcers of the leg which have proved rebellious to all other methods of treatment, can be invariably cured by scraping, followed by the application of iodoform which must be continued for several days, and, after fresh granulations have appeared, by firm pressure with equal parts of mercurial and soap plaster.—*Berl. klin. Wochen.*

---

*Whooping Cough and Ulceration of the Frœnum.*—A recent report before the Academy of Medicine in Paris, says that the ulceration is an affect rather than a cause of the disease, and is due to the protrusion of the tongue and the scraping of its under surface against the teeth, in the act of coughing.—*Med. Record.*

---

*Chloroform Narcosis.*—Wachsmuth, of Berlin, asserts that much of the danger from the administration of chloroform may be averted by adding to it twenty per cent. of oil of turpentine, which, he says, stimulates the lungs, and thus protects them against the great enemy of chloroform narcosis—pulmonary paralysis.—*N. Y. Medical Record.*

---

*A Perfumed Solution of Iodoform.*—Shake tincture of iodine with a fragment of fused potassa till the color be removed. Cover the odor of the iodoform thus produced by the addition of eau de cologne. Dip lint in this solution, allow it to dry, and one will have an agreeable and excellent application for indolent ulcers, fissure ani, burns, etc.—*Med. Record.*

---

*Freckles.*—Take of finely powdered sulphophenate of zinc, one part; oil of lemon, one part; pure alcohol, five parts; collodion, forty-five parts; mix well together by triturition. This has been found efficacious as a local application against freckles and other slight skin diseases.—*Pharm. Zeit. fur Ruos.*

